# Quantifying Podocyte Number in a Small Sample Size of Glomeruli with CUBIC to Evaluate Podocyte Depletion of db/db Mice

**DOI:** 10.1155/2023/1901105

**Published:** 2023-02-01

**Authors:** Jiaoyu Shi, Yuan Hu, Guangze Shao, Yixiang Zhu, Zhonghua Zhao, Yanyong Xu, Zhigang Zhang, Huijuan Wu

**Affiliations:** ^1^Department of Pathology, School of Basic Medical Sciences, Fudan University, Shanghai 200032, China; ^2^Department of Pathology, Zhongshan Hospital, Fudan University, Shanghai 200032, China; ^3^School of Public Health, Key Lab of Public Health Safety of the Ministry of Education, NHC Key Lab of Health Technology Assessment, Fudan University, Shanghai 200032, China; ^4^Key Laboratory of Metabolism and Molecular Medicine of the Ministry of Education, Department of Pathology of School of Basic Medical Sciences, Fudan University, Shanghai 200032, China; ^5^Frontier Innovation Center, School of Basic Medical Sciences, Fudan University, Shanghai 200032, China

## Abstract

The loss of podocyte is crucial for diagnosis and prognosis of diabetic kidney disease, whereas commonly two-dimensional methods for quantifying podocyte number existed with issues of low fidelity and accuracy. In this study, clear, unobstructed brain imaging cocktails and computational analysis (CUBIC), one of three-dimensional optical clearing approaches, was used which combines tissue clearing, immunolabeling, and a light-sheet microscope to image and evaluate podocytes in C57BL/6 (C57) and db/db mice. We discovered that 77 podocytes per glomerulus were in C57 mice. On the subject of db/db mice, there were 74 podocytes by the age of 8 w, 72 podocytes by the age of 12 w, and 66 podocytes by the age of 16 w, compared with 76 podocytes in the control group, suggesting that there was a significant decrease in podocyte number in db/db mice with the age of 16 w, showing a trend which positively correlated to the deterioration of kidney function. Sample size estimation using the PASS software revealed that taking 5%, 7.5%, and 10% of the mean podocyte number per glomerulus as the statistical allowable error and 95% as total confidence interval, 33, 15, and 9 glomeruli were independently needed to be sampled in C57 mice to represent the overall glomeruli to calculate podocyte number. Furthermore, in the control group of db/db mice, 36, 18, and 11 glomeruli were needed, compared with 46, 24, and 14 glomeruli in db/db mice by the age of 8 w, 43, 21, and 12 glomeruli by the age of 12 w, and 52, 27, and 16 by the age of 16 w. These findings indicated that precise quantification of podocyte number could judge the progression of diabetic kidney disease. In addition, a small number of glomeruli could be actually representative of the whole sample size, which indicated apparent practicability of CUBIC for clinical use.

## 1. Introduction

Proteinuria, one of the significant clinical manifestations of diabetic kidney disease (DKD), which could not only lead to edema and hypoalbuminemia but also directly damage renal tubules, is an independent risk factor of end-stage renal disease (ESRD) [1–7]. Occurrence of proteinuria is tightly related to increased permeability of glomerular filtration barrier (GFB) mostly attributed to podocyte loss [8–12]. Podocytes are the vital part of GFB, together with glomerular basement membrane and glomerular endothelial cells, which maintain the filtering capabilities of the kidney [13–16]. When podocytes suffer injury during DKD, they would detach from the glomerular basement membrane and decrease in number [17], which cause compensatory hypertrophy of the remaining podocytes, increased width of podocytes foot process, destruction of GFB, and continuous proteinuria [8, 9]. Therefore, severity of proteinuria is intimately relevant to decreased podocyte number, especially the mean podocyte number per glomerulus, and counting podocyte number is a necessary way for judging the development and prognosis of DKD.

Literatures have reported several ways to count the podocyte number including traditional 2D methods and recently raised 3D methods. The most common method to assess podocyte number is to quantify the podocyte nuclei stained with WT-1, a podocyte nuclear marker, of glomerular cross-sections in kidney tissue slices in two-dimensional (2D) plane [18]. However, recent studies have emphasized multiple limitations of this method, including low fidelity and accuracy [19–21]. Given this, some scholars have developed an approach for counting podocytes by the thick and thin section method [22]. Although this method improves the exactness of counting, the whole process is extremely complicated and would have unavoidable loss of tissue during sectioning, which would result in colossal errors for the final results.

Three-dimensional (3D) optical clearing has received extensive attention for achieving effective 3D visualization and quantification of large tissues and entire organs, which shows magnificent advantages in counting podocyte number [23–26]. At present, there are mainly three kinds of optical clearing including hydrophobic, hydrogel-based, and hydrophilic methods. Clear, unobstructed brain imaging cocktails and computational analysis (CUBIC) is one of hydrophilic optical clearing approaches, which could simply and effectively image with light-sheet microscope and is suitable for multicolor imaging of fluorescent protein or immune-stained samples which involves immersing samples in a chemical mixture containing amino alcohols [27–29]. Compared with other hydrophilic methods such as SeeDB, CUBIC has a faster removal rate, deeper image, and higher transparent effect [30–33]. Moreover, CUBIC shows superior preservation of fluorescent signal compared to hydrophobic methods such as BABB and 3DISCO and would not undergo tissue shrinkage, which often appears in hydrophobic methods and is detrimental for high-resolution imaging [34–36]. In addition, CUBIC is easy to operate, requiring only immerse tissues in several different reagents, rather than dedicated electrophoresis equipment and complicated protocols in hydrogel-based methods such as clarity [37]. Therefore, in the present study, CUBIC was used to quantify the podocytes and glomeruli in both C57 and db/db mice.

However, not like the animal study, only small pieces of renal tissue with only dozens of glomeruli could be sampled by renal biopsy in actual clinical practice, which may cause a miscalculating of the mean podocyte number per glomerulus. For example, in focal segmental glomerular sclerosis, if the actual incidence of infected glomeruli is 10%, there is a 35% probability of missing all diseased glomeruli when a biopsy sample only has 10 glomeruli, and the probability would decrease to 12% when the biopsy contains 20 glomeruli [38]. To achieve the clinical application in the future, we combined the CUBIC methods with appropriate statistical analysis to assess the sample size required calculating the mean podocyte number per glomerulus to represent the whole kidney condition.

Therefore, in this study, we detected podocytes and glomeruli by CUBIC combined with fluorescence staining and light-sheet microscope, and used Imaris software to quantify podocyte number in C57 mice and db/db mice, a diabetic kidney disease model. Furthermore, the number of sampled glomeruli that could represent the overall glomeruli to quantify the podocyte number was analyzed by using the PASS software to discover the possibility of applying this method for judging diagnosis and prognosis of DKD.

## 2. Materials and Methods

### 2.1. Reagents

All the reagents are listed in the Supplementary Table 1 and the detailed documentation of reagent setup is provided in the Supplementary Methods.

### 2.2. Animal Study

Male C57BL/6 (C57) mice, C57BLKS db/db (db/db) mice, and the nondiabetic db/m (the control group) mice were bought from Nanjing Junke biological company. C57 mice and db/m mice are at the age of 8 w and db/db mice are at the age of 8 w, 12 ,w and 16 w. Approval to conduct animal experiments for research purposes (no. 20190221-061; no. 20211020-004) was given by the Ethical Committees of the School of Basic Medical Sciences, Fudan University. All procedures were carried out according to the approved guidelines.

### 2.3. CUBIC Protocol

The detailed CUBIC protocol is shown in the Supplementary Figure 1.

### 2.4. 3D Quantification of Podocyte

Podocytes were defined as WT-1-stained cells and turned to spheres after 3D reconstruction by Imaris, version 9.0 (Bitplane AG) for quantification. Quantification of glomerular volume was also conducted by using 3D rendering with Imaris. Individual glomerulus was separated by a 3D crop panel.

### 2.5. Kidney Histology and Immunohistochemistry

Mice kidneys were fixed in 4% paraformaldehyde for paraffin-embedded kidney sections (3 *μ*m), which were then deparaffinized and rehydrated for the following staining techniques. For kidney histologic examination, periodic acid–Schiff (PAS) staining was performed using the standard methods. For immunohistochemistry, 3% H2O2 was used to remove endogenous peroxidase, and the antigen was retrieved in Tris-EDTA (TE) buffer, following by blocking with 5% normal goat serum and incubating with the primary antibody at 4°C overnight (WT-1: 1 : 100). After washing three times in PBS, the sections were then incubated with the appropriate secondary antibodies for 1 h in room temperature, washed with PBS three times again, and the signals were visualized using liquid 3,3'-Diaminobenzidine (DAB) + substrate chromogen system (Dako, USA), followed by counterstaining with hematoxylin and capturing images using a multiple viewing microscope (Nikon).

### 2.6. Measurement of Kidney Function

The urine samples were collected from db/db mice at different ages indicated in the corresponding figure, and the urine albumin-to-creatinine ratio (uACR) was immediately measured at Shanghai General Hospital.

### 2.7. Statistical Analyses

Statistical analyses were performed by using GraphPad Prism version 9.0 (GraphPad Software, San Diego, CA, USA) and PASS (Power Analysis and Sample Size) 11.0. Comparisons between two subgroups were carried out via an unpaired *t*-test. Quantitative parameters were shown as the mean ± standard error of the mean. We used Pearson's correlation to assess the association between the decrease in podocyte per glomerulus and age of db/db mice. *P* values < 0.05 were considered statistically significant compared with control.

## 3. Results and Discussion

### 3.1. Results

#### 3.1.1. CUBIC, a Convenient Method to Image Glomeruli and Podocytes

**Figure 1:**
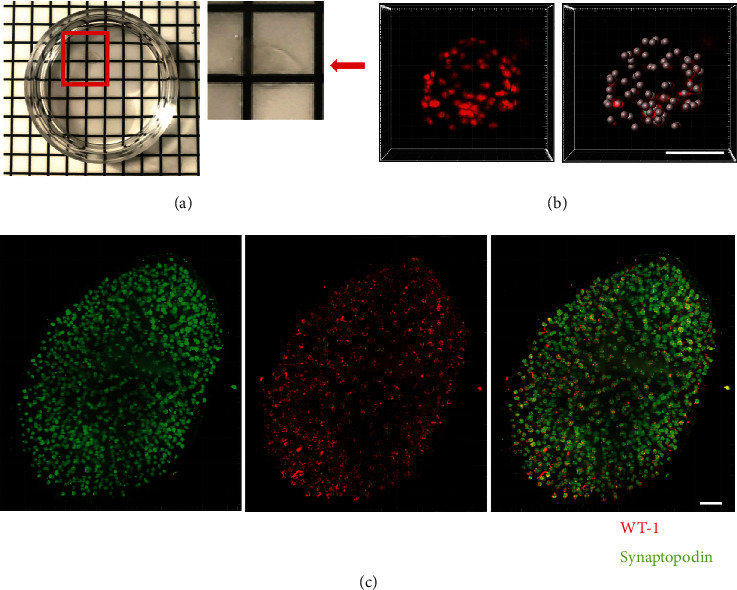
Optical clearing, light-sheet microscope, and 3D rendering for whole glomeruli. (a) 2 mm thick kidney tissue from C57 mice was analyzed by optical clearing using CUBIC method. Red arrow displayed the location of the translucent tissue. (b) Double immunofluorescence staining with podocyte cytoplasmic marker synaptopodin (green) and podocyte nuclear marker WT-1 (red) identified glomeruli and podocytes with light-sheet microscope by using the CUBIC method (screenshots from three-dimensional video). (c) Podocyte nuclei stained with WT-1 (red) of a single whole glomerulus and the linked 3D reconstruction image are shown, which depicted the practicality of counting podocyte number with the CUBIC method. Shown are representative images from five C57 mice with similar results (scale bar: 50 *μ*m in all images of (b); 400 *μ*m in all images of (c)).

CUBIC is a common method for optical clearing, which could quickly get three-dimensional images with fewer equipment and reagents and easy procedure, and it could preserve fluorescent signals for several days. To completely image numerous whole glomeruli and podocytes, 2 mm thick kidney tissues of C57 mice were treated by CUBIC as protocol in Materials and methods. After optical clearing, the whole kidney section transformed to be translucent (Figure 1(a)).

The kidney section was stained with WT-1 (podocyte nuclear marker in red) and synaptopodin (podocyte cytoplasmic marker in green), which were used to identify glomeruli and podocytes with light-sheet microscope (Figure 1(b) and Supplementary Video). WT-1-stained nuclei could identify the podocyte nuclei, which could be quantified through 3D reconstruction by the Imaris software (Figure 1(c)).

#### 3.1.2. Podocytes per Glomerulus Calculated from a Small Number of Glomeruli May Represent That from the Overall Glomeruli in C57 Mice

**Figure 2:**
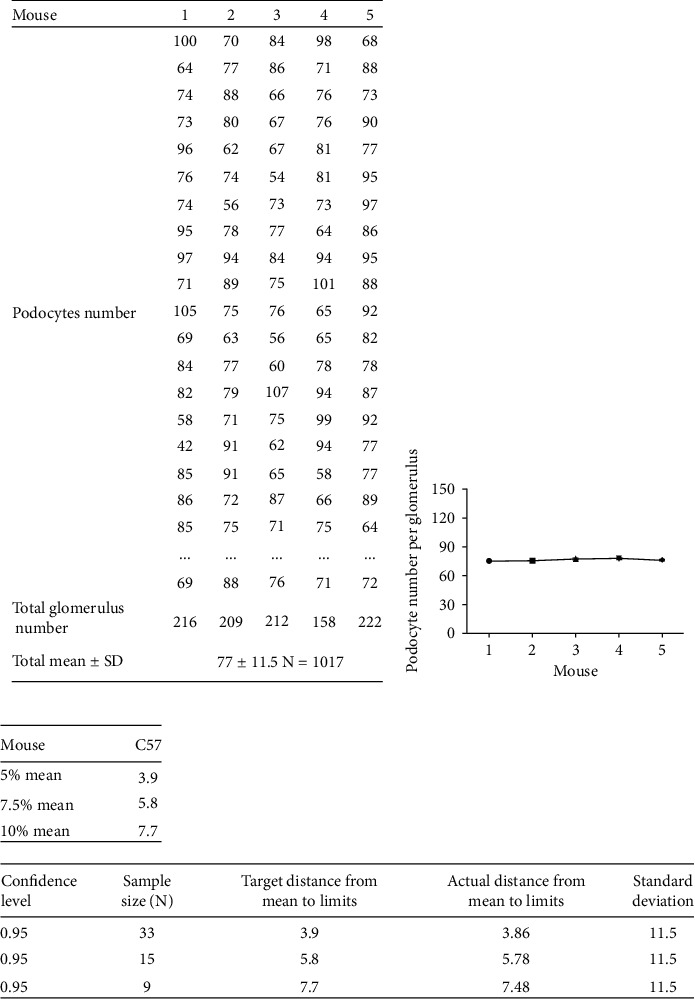
A limited number of glomeruli may represent the overall podocyte number in C57 mice. (a) The podocyte number per glomerulus of 1017 glomeruli in five C57 mice was quantified with the Imaris software through identifying 3D reconstruction images of podocytes per glomerulus and exhibited in chart. The mean of podocyte number per glomerulus was calculated from 1017 glomeruli. The sum of podocyte number from 1017 glomeruli was used as the numerator, which was divided by the total number of glomeruli, 1017. (b) No statistical significance in podocyte number per glomerulus was observed among five C57 mice, which proposed stability distribution of podocyte number per glomerulus within C57 mice. Data are expressed as mean ± SEM. (c) The tables displayed the sample size of glomeruli needed to represent the whole sample size with taking 5%, 7.5%, and 10% of the mean podocyte number per glomerulus as the statistical allowable error and setting total confidence level as 95% by the PASS software.

The podocyte number in C57 mice was quantified through 3D reconstruction of WT-1-stained nuclei from 1017 glomeruli. The total podocyte number per glomerulus in C57 mice ranged from 42 to 117 (mean = 77 ± 11.5) (Figures 2(a) and 2(b)), which was similar with the findings from Puelles et al. showing 71.7 ± 16.2 per glomerulus of mice with unknown background [24].

If a novel technology could be used in clinical, it would be of great value. Therefore, to investigate the possibility of CUBIC combined with 3D imaging in the application of renal disease diagnosis and estimation of prognosis, the PASS software was used to figure out how much glomeruli sampled could represent the whole sample size, since only limited glomeruli could get by biopsy clinically. Setting total confidence level as 95% and taking 5% of the mean podocyte number per glomerulus as the statistical allowable error, then 33 glomeruli are needed to represent the overall glomeruli in C57 mice for calculating the podocyte number per glomerulus. Taking 7.5% of the mean podocyte number per glomerulus as the statistical allowable error, 15 glomeruli are requested. And only 9 glomeruli are needed to be sampled, when the statistical allowable error was 10% of the mean podocyte number per glomerulus (Figure 2(c)).

#### 3.1.3. Definition of Absolute Podocyte Decrease in db/db Mice

**Figure 3:**
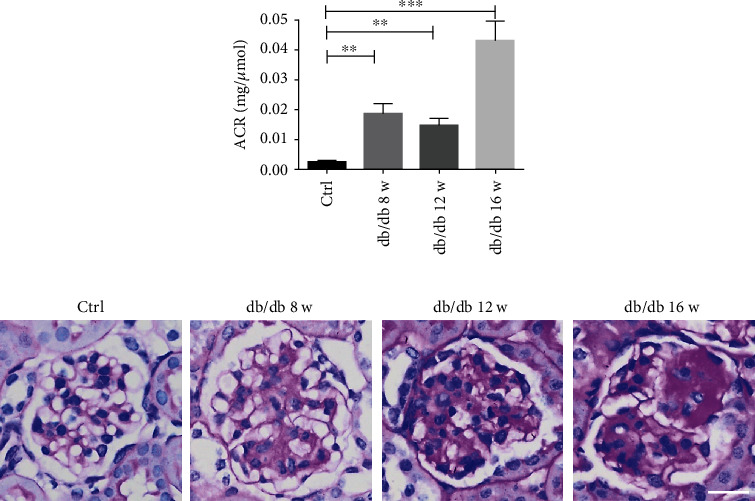
Characterization of db/db mice. (a) db/db mice (*n* ≥ 3) had significantly increased urine ACR compared with the control group (*n* = 4). Data are expressed as mean ± SEM; ^∗∗^*P* < 0.01, ^∗∗∗^*P* < 0.001 for db/db mice by the age of 8 w, 12 w, and 16 w versus the control group. (b) PAS-stained kidney sections revealed increased glomerular mesangial matrix in db/db mice compared with the control group. Shown are representative images from at least three mice per group with similar results (scale bar: 100 *μ*m in all images of (b)).

Then db/db mice were selected to estimate the value of both CUBIC and sample size analysis in the actual application of disease diagnosis. Since the occurrence of proteinuria is closely related to improved permeability of glomerular filtration membrane mostly caused by podocyte loss, examining the change of podocyte number is significant for judging the course of diabetic kidney disease. By urine albumin-to-creatinine ratio (uACR) detection and periodic acid–Schiff (PAS) staining, we confirmed the establishment of diabetic kidney disease model with a slight increase in uACR of db/db mice by the age of 8 w and 12 w and a remarkable increase by the age of 16 w, compared to the control group (Figure 3(a)). Additionally, glomerular mesangial matrix was slightly increased in db/db mice by the age of 8 w and 12 w and remarkably increased by the age of 16 w with formation of the K-W nodule (Figure 3(b)).

**Figure 4:**
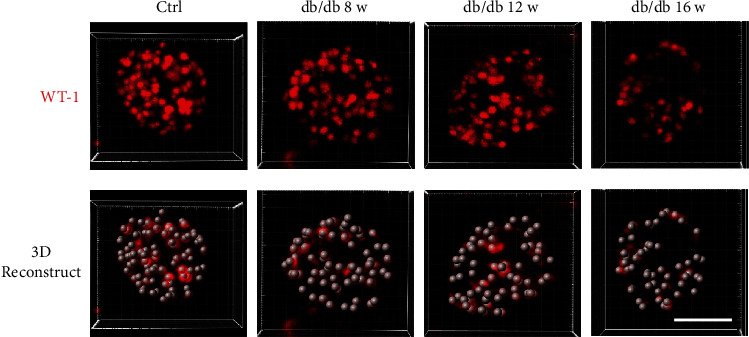
Three-dimensional visualization of podocytes in control and DKD glomeruli. Representative images of podocytes from the control group and db/db mice labeled with antibody to WT-1 (red) by the CUBIC method. Podocytes are visible as higher density of WT-1-stained nuclei is observed within the control group than db/db mice. Shown are representative images from three mice of every group with similar results (scale bar: 50 *μ*m).

**Figure 5:**
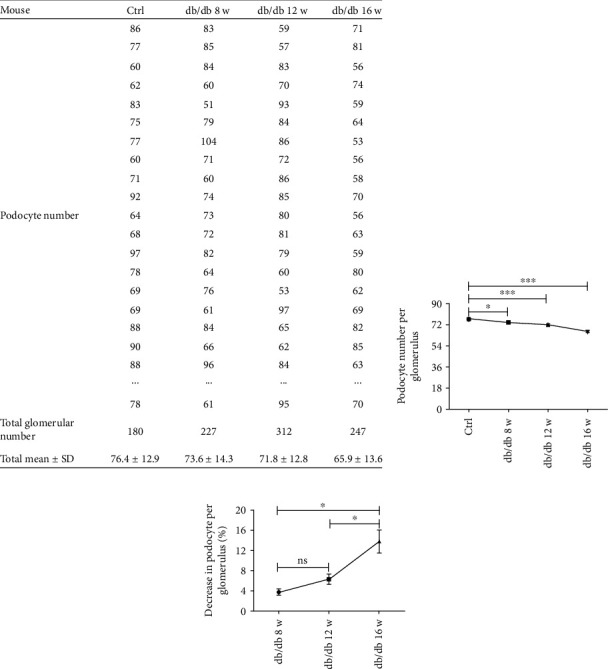
Absolute podocyte decrease of whole glomerulus in db/db mice. (a) The tabulation manifested podocyte number per glomerulus of the control group and db/db mice (three mice per group). The total podocyte number per glomerulus of every group was calculated through sum of all podocyte number divided by glomerular number of each group. (b) Statistically significant decrease in podocyte number per glomerulus in db/db mice indicated a more severe renal injury. Data are expressed as mean ± SEM. ^∗^*P* < 0.05, ^∗∗∗^*P* < 0.001, and ^∗∗∗∗^*P* < 0.0001 for db/db mice by the age of 8 w, 12 w, and 16 w versus the control group. (c) The decreased rate of podocyte number was expressed more noticeable in db/db mice in age of 16 w, compared with those in age of 8 w and 16 w. The decreased rate of podocyte number was calculated through the difference of mean podocyte number per glomerulus between each db/db mice (at least 70 glomeruli per mouse) and the control group divided by mean podocyte number per glomerulus in the control group (180 glomeruli). Data are expressed as mean ± SEM. ^∗^*P* < 0.05, ns: no significance.

By comparing the 3D reconstruction image of podocyte (Figure 4) and statistical analysis of the podocyte number between the control group and the db/db mice with age of 8 w, 12 w, or 16w, the total podocyte number per glomerulus was found ranging from 46 to 115 (mean = 76.4 ± 12.9) in the control group, whereas the podocyte number per glomerulus exhibited a downward trend in db/db mice showing 42 to 110 (mean = 73.6 ± 14.3) at 8 w of age, 34 to 104 (mean = 71.8 ± 12.8) at 12 w of age, and 24 to 99 (mean = 65.9 ± 13.6) at 16 w of age (Figures 5(a) and 5(b)). Although there was no significant difference of the decreased rate of podocyte number between the ages of 8 w and 12 w, a notable reduction was observed in age of 16 w (Figure 5(c) and Supplementary Figure 3), which was consistent with the decreasing trend of uACR, suggesting a strong relationship between podocyte number and proteinuria.

#### 3.1.4. Podocyte Number in the Context of Glomerular Volume Is Deceased

**Figure 6:**
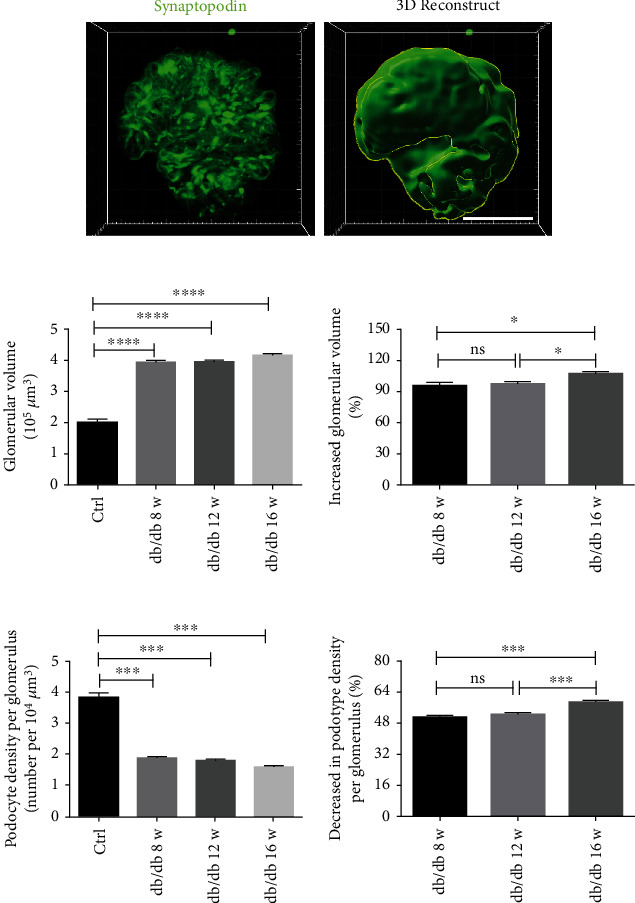
Relative podocyte decrease of whole glomerulus in db/db mice. (a) Three-dimensional visualization of podocytes labeled with synaptopodin was expanded to obtain a 3D reconstruction of the whole glomerulus as glomerular volume (scale bar, 50 *μ*m). (b) db/db mice had significantly increased glomerular volume, compared with the control group (at least 80 glomeruli per group), which there was a more marked increase in db/db mice with age of 16 w. The increased glomerular volume rate was calculated through the difference of mean glomerular volume between each db/db mice (at least 25 glomeruli per mouse) and the control group divided by mean glomerular volume in the control group (80 glomeruli). Data are expressed as mean ± SEM; ^∗^*P* < 0.05, ^∗∗∗∗^*P* < 0.0001. (c) A statistically significant decrease in podocyte density per glomerulus in db/db mice with a remarkable decrease podocyte density per glomerulus by the age of 16 w was presented by column diagrams. Podocyte density per glomerulus of each group was calculated through mean total podocyte number per glomerulus divided by glomerular volume. Decrease in podocyte density per glomerulus of each group was calculated through difference of podocyte density per glomerulus between each db/db mice and the control group divided by mean podocyte density per glomerulus in the control group. Data are expressed as mean ± SEM; ^∗∗∗^*P* < 0.001.

In response to the state of high filtration during the course of DKD, glomeruli would undergo structural and functional changes, such as glomeruli hypertrophy, which could lead to meaningful variation in podocyte density caused by alternation of glomerular volume. Glomerular volume was estimated by morphologic dilation on Imaris using synaptopodin as a cytoplasmic marker of podocyte and expanding stained area to obtain a 3D reconstruction of the whole glomerulus (Figure 6(a)). db/db mice developed substantially increased glomerular volume compared with the control group, of which there was an equivalent raise between db/db mice by the age of 8 w and 12 w, and a significant increase by the age of 16 w (Figure 6(b)). We also calculated podocyte density per glomerulus of each group through mean total podocyte number per glomerulus divided by glomerular volume, which demonstrated a more obvious diminish of db/db mice in the age of 16 w, with an equal decline between db/db mice in ages of 8 w and 12 w (Figure 6(c)).

#### 3.1.5. Podocytes per Glomerulus Calculated from a Small Number of Glomeruli May Represent That from the Overall Glomeruli in db/db Mice

**Figure 7:**
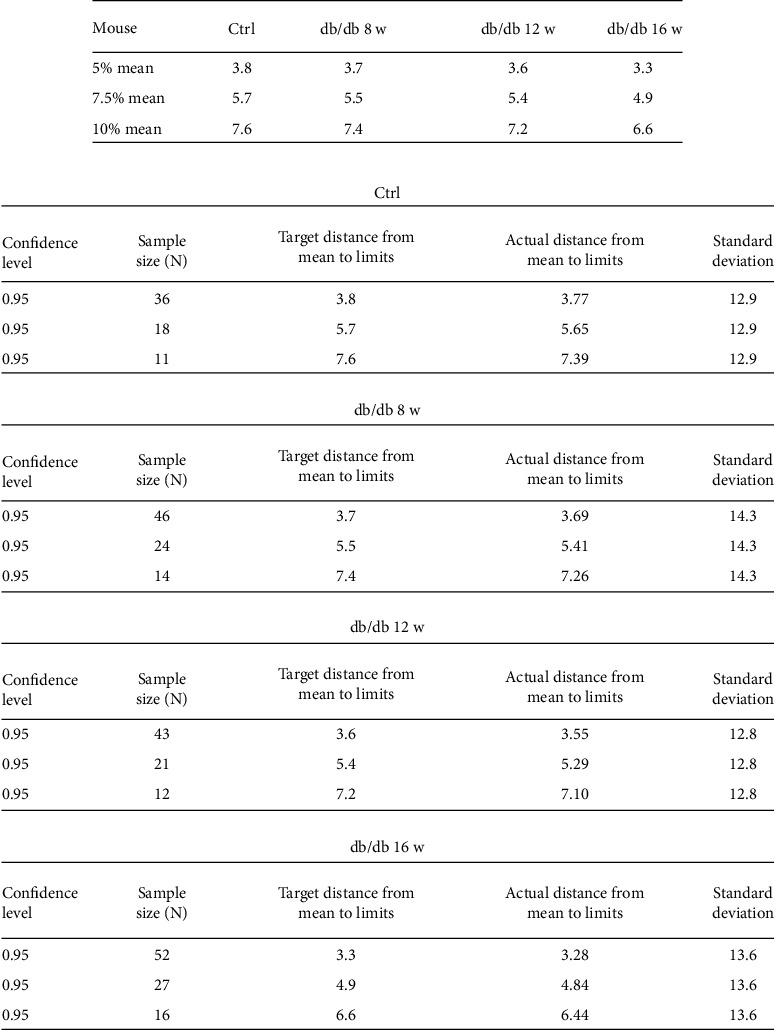
On behalf of the entire podocyte number, a slight number of glomeruli were required in db/db mice. Taking (a) 5%, 7.5%, and 10% of the mean number of podocytes per glomerular as the statistical allowable error with total confidence level as 95%, (b) the corresponding sample size of the control group and db/db mice by the age of 8 w, 12 w, and 16 w was separately presented in the table by using the PASS software.

To verify whether limited glomeruli sample could represent the whole glomeruli sample size to calculate the podocytes per glomerulus in db/db mice, the PASS software was utilized to find out the necessary number of glomeruli required in every group of db/db mice. In the control group, 36 glomeruli are needed to represent the overall glomeruli in db/db mice for calculating the podocyte number per glomerulus by setting total confidence level as 95% and 5% of the mean podocyte number per glomerulus as the statistical allowable error, compared with 18 and 11 glomeruli when the statistical allowable error was 7.5% and 10% of the mean podocyte number per glomerulus separately. In db/db mice by the age of 8 w, 46, 24, and 14 glomeruli were individually needed with 5%, 7.5%, and 10% of the mean podocyte number per glomerulus as the statistical allowable error, while 43, 21, and 12 glomeruli were needed in db/db mice by the age of 12 w, with 52, 27, and 16 required in db/db mice by the age of 16 w (Figure 7). All above suggested feasibility of the method for the clinical use.

## 4. Discussion

Accurately quantifying podocyte number is extremely significant in assisting the diagnosis of kidney diseases and guiding clinical medication especially in diabetic kidney disease, focal segmental glomerulosclerosis (FSGS), and other podocyte injury diseases with nephrotic syndrome or nephrotic range proteinuria as the main manifestation, because of the pivotal role of podocytes in maintaining glomerular filtration barrier's structure and preventing the occurrence of proteinuria [15, 16, 39–43]. Due to limitations of counting podocyte number in 2D plane [19–21], we quantified podocyte number with CUBIC, one of 3D tissue optical clearing methods, which have showed great advance in counting podocyte number on account of simple protocol, splendid preservation of fluorescent signal, no tissue shrinkage, and higher transparent effect compared with other optical clearing methods [27–37]. In our study, the podocyte number per glomerulus of C57 mice was about 77, which was basically consistent with 72 reported in unknown background mice by the BABB method [25], highlighting the feasibility of the CUBIC method.

As shown in “Supplementary Figure 2a-b”, we also examined the podocyte number per glomerular cross-section quantified by WT-1 immunohistochemistry staining in 2D plane and found that compared to the control group, the significant decrease in podocyte number of db/db mice could be observed from the age of 12 w and 16 w whereas no obvious change of podocyte number could be detected by the age of 8 w. However, by using the CUBIC method in this study, not only by the ages of 12 w and 16 w but also by the age of 8 w, the decrease of both absolute and relative podocyte number per glomerulus in db/db mice was observed compared with the control group. Actually, the uACR has already increased with increased mesangial matrix in db/db mice by the age of 8 w, which matches the decreased podocyte number at 8 w quantified by the CUBIC method rather than the 2D methods. Therefore, quantifying podocyte with CUBIC is more accurate than traditional 2D methods, since it could detect minor differences among db/db mice with a different age, and furthermore, the change of podocyte number was closely and positively related to the severity of proteinuria. Due to the crucial role of the podocyte in proteinuria [44] and intimate relationship between podocyte number and progression of proteinuria in diabetic kidney disease, FSGS, IgA nephropathy, and other kidney diseases [45–49], it would be of great help in judging the course of those glomerulonephritis showing proteinuria by quantifying decrease in podocyte number with CUBIC.

The sample size of glomeruli is a substantial issue that should be considered for the renal pathologists when using CUBIC in clinical in the future, because there would not be such a huge number of glomeruli obtained from biopsy to quantify. As we know, if the number of glomeruli is below a threshold value, the accuracy of the observation of a focal lesion and the diagnosis of the renal disease will be affected. The well-known example in renal biopsy is focal segmental glomerular sclerosis, and usually 25, glomeruli are needed to get a relatively correct diagnosis, since there was a 12% possibility to miss the focal lesion within glomeruli if a biopsy had only 20 glomeruli [38]. As we showed in the study, the podocyte number either in C57 mice or in db/db mice shows a wide number range with 42 as the lowest and 117 as the highest, which could mislead the evaluation if only a tiny biopsy tissue with limited glomeruli was sampled. Therefore, we used the PASS software to find out how much glomeruli should be sampled to represent the overall podocyte number level. When taking 5%, 7.5%, and 10% of the mean podocyte number per glomerulus as the statistical allowable error with total confidence interval of 95%, there were 33, 15, and 9 glomeruli to be sampled in C57 mice, respectively. And for the DKD animal model, in the control group, there are 36, 18, and 11 glomeruli needed to represent the overall glomeruli to calculate the podocyte number per glomerulus, compared with 46, 24, and 14 glomeruli in db/db mice by the age of 8 w, 43, 21, and 12 glomeruli with the age of 12 w and 52, 27, and 16 in the age of 16 w, which answered an important question that how small the sample size required by biopsy could be actually representative of the whole sample size, thus indicating the value of CUBIC combining with statistical method for clinical use.

The CUBIC process was conducted as SUSAKI's article exhibited [27], during which there were several experiment points need to be highlighted in our study for better transparent effect and more clearly fluorescent signal. Firstly, fixing time with paraformaldehyde (PFA) would affect fluorescent staining effect of tissues, once which was more than two weeks, and there were no effective fluorescent signal that could be detected by our observation. Besides, ambient condition of experimental room was equally important for success of CUBIC in consequence that antibodies for fluorescent staining should be shaken in constant temperature shaker at 37°C and would be spoiled when weather turned to be muggy. Therefore, the temperature of experimental room should be controlled around 25°C; otherwise, antibodies get spoiled easily during the course. Furthermore, compared with immersing tissue for two days in reagent-2 as described in supplementary methods, immersing tissue for five days in reagent-2 would bring a better transparent effect with less swelling.

In summary, our study indicates that CUBIC is an excellent method to detect podocyte number in those institutions or organizations with appropriate devices including light-sheet or confocal microscope, which can predict progression of proteinuria. And in order to make the CUBIC method available for clinical use, this study demonstrates for the first time how much glomeruli need to be sampled to represent the overall glomeruli to calculate podocyte number in C57 mice and db/db mice, which establishes the practicality of the CUBIC method for judging diagnosis and prognosis of DKD.

## 5. Conclusions

Quantifying the podocyte number precisely could judge progression of diabetic kidney disease. In addition, a small number of glomeruli could be actually representative of the whole sample size, which indicated apparent practicability of CUBIC for clinical use.

## Data Availability

The data used to support the findings of this study are available from the corresponding authors upon reasonable request.

## Supplementary Materials

Supplementary Materials Table S1: all the reagents needed. Figure S1: every step of CUBIC. Figure S2: podocyte number per glomerular cross-section decreased in db/db mice by the age of 12 w and 16 w. (a) The diagram displayed podocyte number per glomerular cross-section of the control group and db/db mice (three mice per group), which was detected by WT-1 staining. The total podocyte number per glomerular cross-section of every group was calculated through the sum of all podocyte number divided by glomerular cross-section number of each group. (b) Statistically significant decrease in podocyte number per glomerular cross-section occurred in db/db mice with the age of 12 w and 16 w. Data are expressed as mean ± SEM. ns: no significance, ^∗∗∗∗^*P* < 0.0001 for db/db mice at the ages of 8 w, 12 w, and 16 w versus the control group. Figure S3: Pearson's correlation analysis between podocyte depletion and age of db/db mice. Supplementary Video: three-dimensional video of double immunofluorescence staining with podocyte cytoplasmic marker synaptopodin (green) and podocyte nuclear marker WT-1 (red) identified glomeruli and podocytes with light-sheet microscope by using CUBIC method. Supplementary Methods: all reagent setup needed.
