# Supplementary material for: Quantifying Podocyte Number in a Small Sample Size of Glomeruli with CUBIC to Evaluate Podocyte Depletion of db/db Mice
Source: J Diabetes Res. 2023 Feb 1;2023:1901105. doi: 10.1155/2023/1901105 (PMC9908347; doi:10.1155/2023/1901105)
Supplement: Supplementary Materials — Table S1: all the reagents needed. Figure S1: every step of CUBIC. Figure S2: podocyte number per glomerular cross-section decreased in db/db mice by the age of 12 w and 16 w. (a) The diagram displayed podocyte number per glomerular cross-section of the control group and db/db mice (three mice per group), which was detected by WT-1 staining. The total podocyte number per glomerular cross-section of every group was calculated through the sum of all podocyte number divided by glomerular cross-section number of each group. (b) Statistically significant decrease in podocyte number per glomerular cross-section occurred in db/db mice with the age of 12 w and 16 w. Data are expressed as mean ± SEM. ns: no significance, ∗∗∗∗P < 0.0001 for db/db mice at the ages of 8 w, 12 w, and 16 w versus the control group. Figure S3: Pearson's correlation analysis between podocyte depletion and age of db/db mice. Supplementary Video: three-dimensional video of double immunofluorescence staining with podocyte cytoplasmic marker synaptopodin (green) and podocyte nuclear marker WT-1 (red) identified glomeruli and podocytes with light-sheet microscope by using CUBIC method. Supplementary Methods: all reagent setup needed. [file 1901105.f1.docx]

**Supplementary Table 1**

**
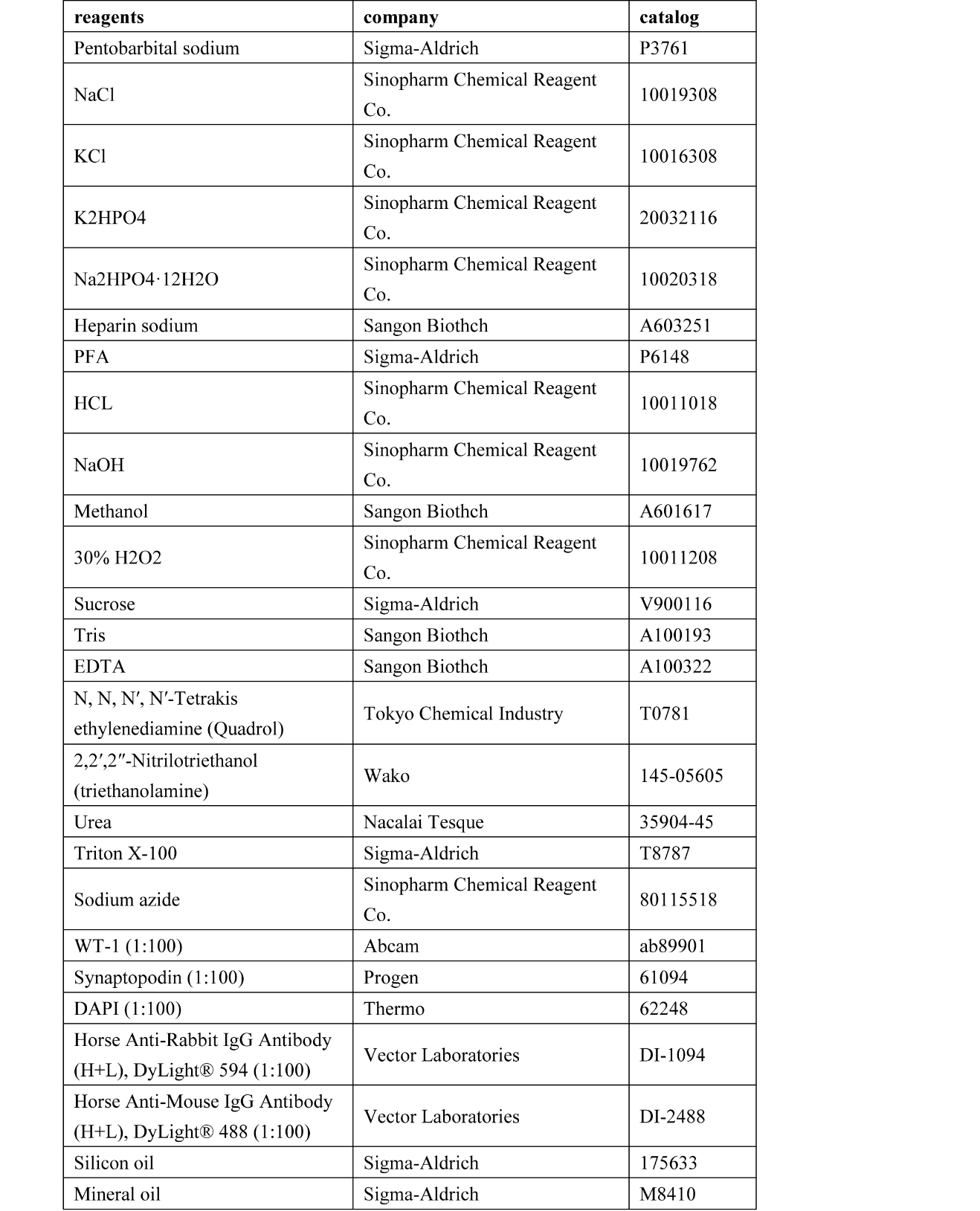
**

**Supplementary Table 1.** All the reagents needed are listed.

**Supplementary Figure 1**


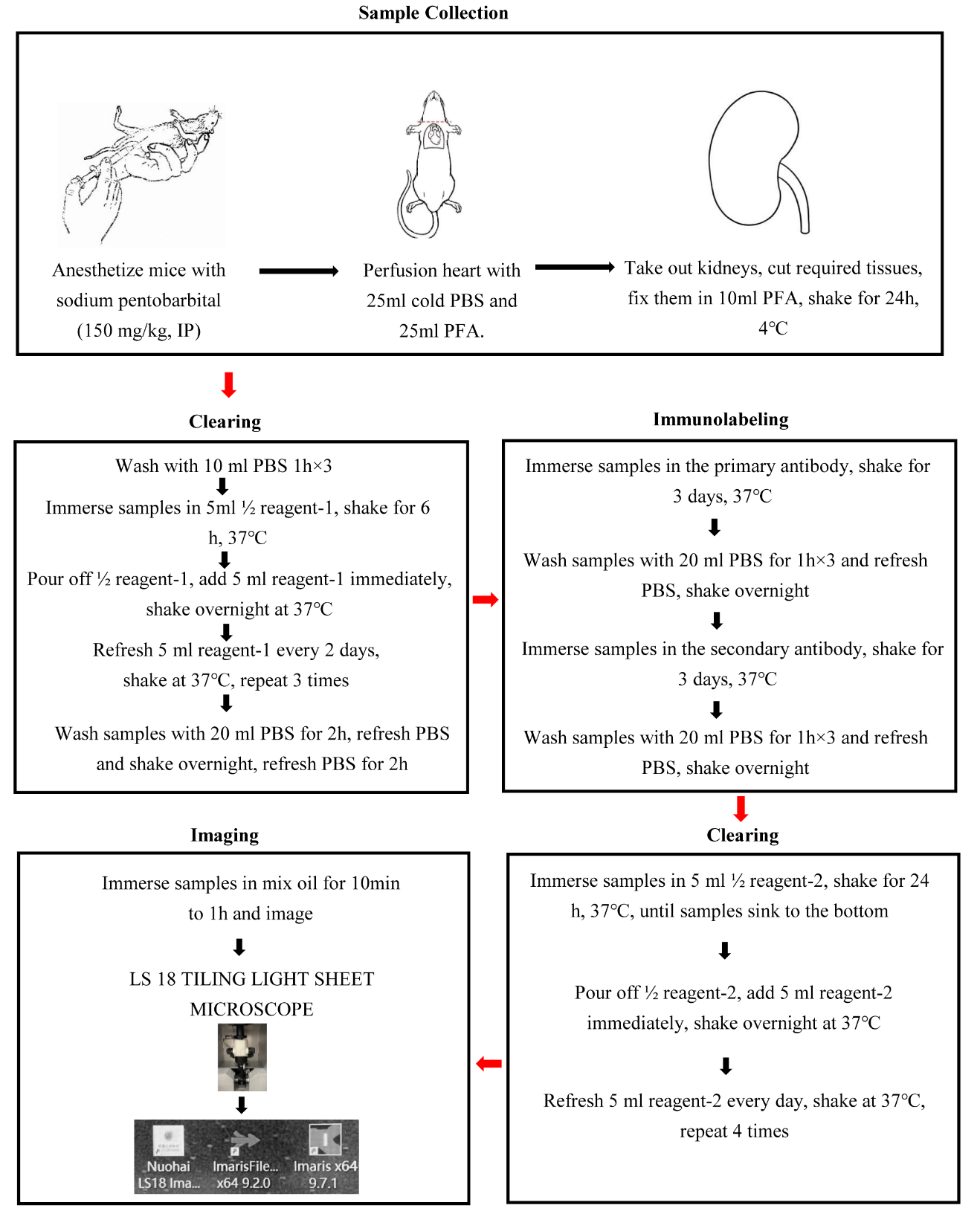


**Supplementary Figure 1.** CUBIC protocol. Every step of CUBIC is explicitly listed in the graph.

**Supplementary Figure 2**


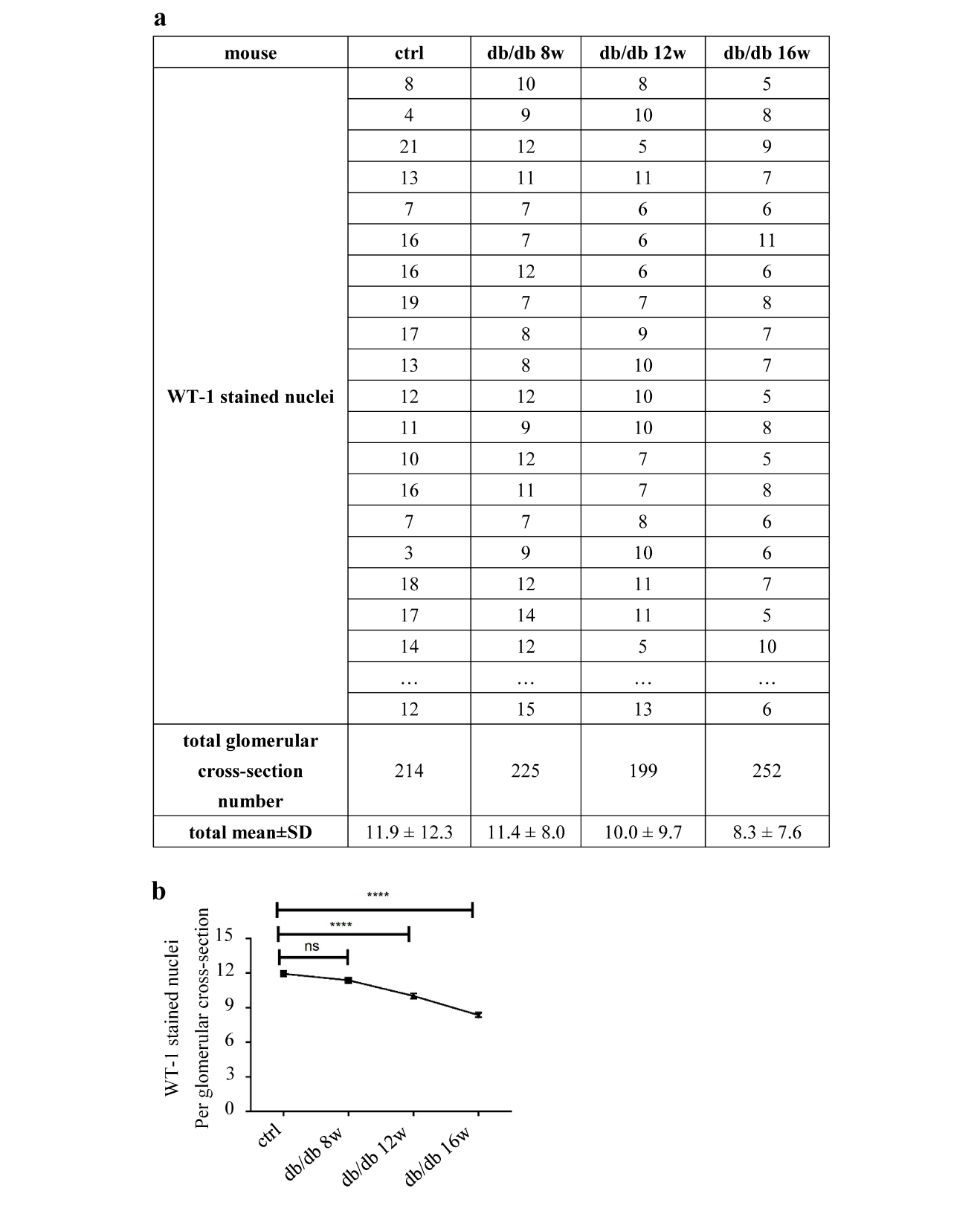


**Supplementary Figure 2.** Podocyte number per glomerular cross-section decreased in db/db mice by the age of 12w and 16w. (a) The diagram displayed podocyte number per glomerular cross-section of the control group and db/db mice (three mice per group), which was detected by WT-1 staining. Total podocyte number per glomerular cross-section of every group was calculated through sum of all podocyte number divided by glomerular cross-section number of each group. (b) Statistically significant decrease in podocyte number per glomerular cross-section occurred in db/db mice with the age of 12w and 16w. Data are expressed as mean±SEM. ns=no significance, ****P<0.0001 for db/db mice at the age of 8w,12w and 16w versus the control group.

**Supplementary Figure 3**


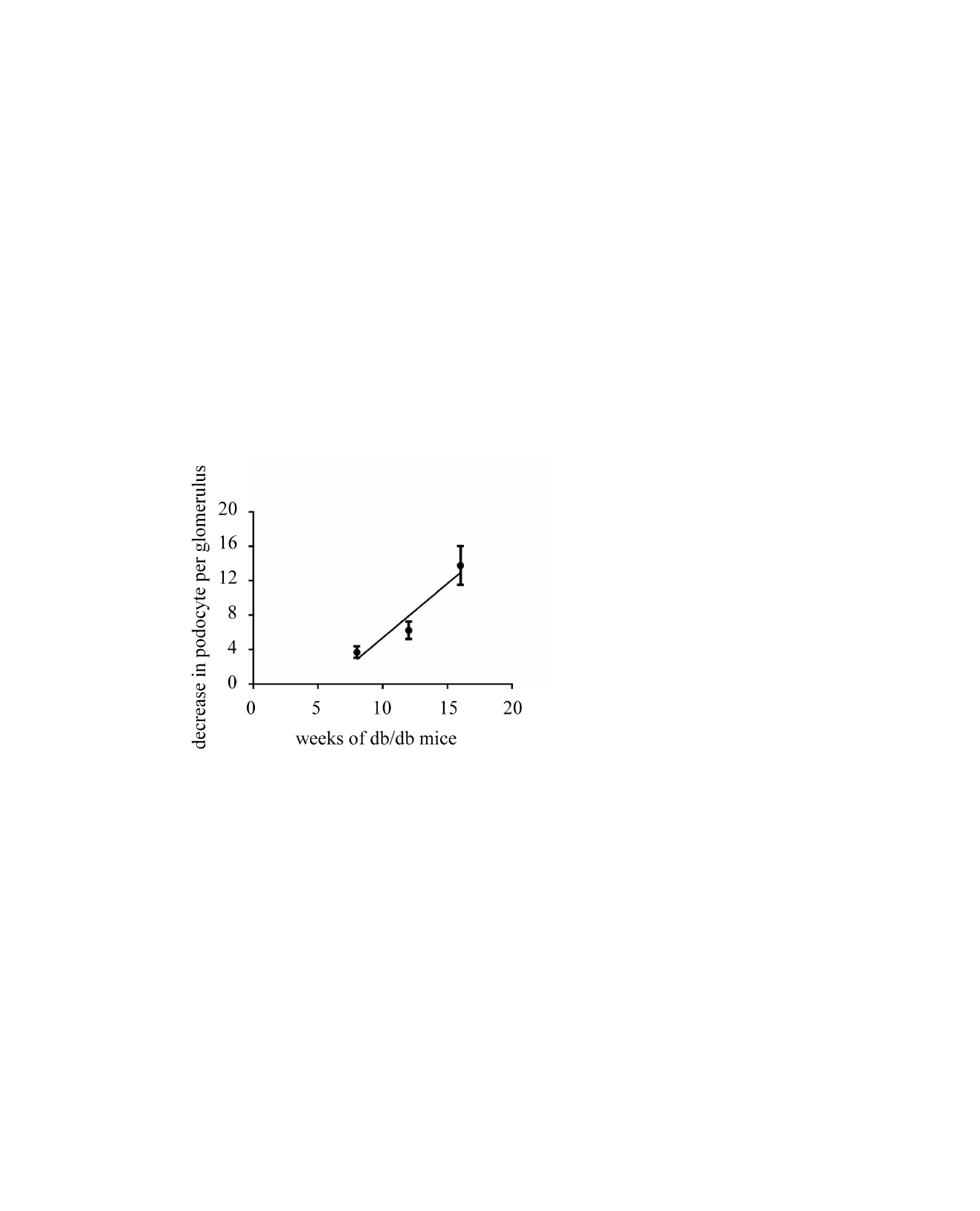


Supplementary Figure 3. Pearson Correlation Analysis between podocyte depletion and age of db/db mice. Linear regression analysis showed there was a close relationship between podocyte depletion and age of db/db mice (R2: 0.7469, P=0.0027).

**Supplementary Video**

**Supplementary Video.** Three-dimensional video of double immunofluorescence staining with podocyte cytoplasm marker synaptopodin (green) and podocyte nuclear marker WT-1 (red) identified glomeruli and podocytes with light-sheet microscope by using CUBIC method.

**Supplementary Methods**

**Reagent setup**

**PBS**

To prepare PBS, dissolve 8g NaCl, 0.2g KCl, 0.2g K2HPO4 and 3.48g Na2HPO4·12H2O in 1L of ddH2O. To prepare PBS/0.01% sodium azide, dissolve 0.1g sodium azide directly in 1L of PBS.

**PFA solution**

To prepare 4% PFA, dissolve 40g PFA powder in 1L of PBS. Heating the PBS solution (avoid boiling) and adding PFA powder and 1/500-1/1,000 volume of 1 N NaOH to accelerate the dissolution of PFA. After complete dissolution, adjusting the pH to 7.4 with HCl.

**80 wt% Quadrol**

Quadrol is a high viscosity liquid and can prepare as an 80 wt% working solution to use. In this case, add 125g ddH2O to 500g Quadrol and stir for at least 30 minutes.

**Reagent-1**

Reagent-1 is a mixture of urea (25wt% final concentration), Quadrol (25wt% final concentration), Triton X-100 (15wt% final concentration) and ddH2O. For example, to prepare 500g reagent-1, mix 125g urea and 156g 80wt% Quadrol in 144g ddH2O using a hot stirrer. After complete dissolution, the mixture is cool at room temperature, then adds 75g Triton X-100. Finally, let it stand overnight to remove air bubbles. Prepare 1/2 reagent-1 by mixing 1:1 reagent-1 and ddH2O.

**Reagent-2**

Reagent-2 is a mixture of urea (25wt% final concentration), sucrose (50wt% final concentration), triethanolamine (10wt% final concentration), and ddH2O. To prepare 50g reagent-2, dissolve 12.5g urea and 25g sucrose in 7.5g ddH2O using a hot stirrer. After complete dissolution, the mixture is cool at room temperature, then add 5g of triethanolamine and continue stirring. Finally, let it stand overnight to remove air bubbles. Prepare 1/2 reagent-2 by mixing 1:1 reagent-2 and PBS.

**Immersion oil mix**

Before use, thoroughly mix silicone oil and mineral oil at 1:1 and let it stand overnight to remove bubbles.

**The primary and the second antibody dilute solution**

Moderate PBS solution containing 0.1% Triton X-100, 0.5% bovine serum albumin and 0.01% sodium azide solution.

**3% H2O2**

3% H2O2 is composed with 5ml 30% H2O2 and 45ml methanol.

**TE buffer**

To prepare TE buffer, dissolve 1.21g Tris and 0.37g EDTA in 1 L of DDW, and adjust the pH to 9.0.

戊巴
